# A chimeric thermostable M2e and H3 stalk-based universal influenza A virus vaccine

**DOI:** 10.1038/s41541-022-00498-6

**Published:** 2022-06-29

**Authors:** Jeeva Subbiah, Judy Oh, Ki-Hye Kim, Chong-Hyun Shin, Bo Ryoung Park, Noopur Bhatnagar, Baik-Lin Seong, Bao-Zhong Wang, Sang-Moo Kang

**Affiliations:** 1 Center for Inflammation, Immunity & Infection, Institute for Biomedical Sciences, Georgia State University, Atlanta, GA 30303 USA; 2 Department of Microbiology, College of Medicine, Yonsei University, 50 Yonsei-ro, Seodaemun-gu, Seoul, 03722 Republic of Korea; 3 Vaccine Innovative Technology ALliance (VITAL)-Korea, Yonsei University, 50 Yonsei-ro, Seodaemun-gu, Seoul, 03722 Republic of Korea

**Keywords:** Protein vaccines, Viral infection

## Abstract

We developed a new chimeric M2e and H3 hemagglutinin (HA) stalk protein vaccine (M2e-H3 stalk) by genetic engineering of modified H3 stalk domain conjugated with conserved M2e epitopes to overcome the drawbacks of low efficacy by monomeric domain-based universal vaccines. M2e-H3 stalk protein expressed and purified from *Escherichia coli* was thermostable, displaying native-like antigenic epitopes recognized by antisera of different HA subtype proteins and influenza A virus infections. Adjuvanted M2e-H3 stalk vaccination induced M2e and stalk-specific IgG antibodies recognizing viral antigens on virus particles and on the infected cell surface, CD4^+^ and CD8^+^ T-cell responses, and antibody-dependent cytotoxic cell surrogate activity in mice. M2e-H3 stalk was found to confer protection against heterologous and heterosubtypic cross-group subtype viruses (H1N1, H5N1, H9N2, H3N2, H7N9) at similar levels in adult and aged mice. These results provide evidence that M2e-H3 stalk chimeric proteins can be developed as a universal influenza A virus vaccine candidate for young and aged populations.

## Introduction

Influenza virus transmissions can be controlled by effective vaccination. However, the effectiveness of influenza vaccination inducing neutralizing antibodies to strain-specific hemagglutinin (HA) proteins is low due to constant antigenic changes in the HA1 receptor-binding globular head domain, rendering pre-existing immunity ineffective to new pandemics. For example, during the 2014–2015 season, drifting mutations in circulating H3N2 strains significantly reduced the effectiveness to 6% against H3N2 subtype virus^1^. Influenza A virus HA subtypes are phylogenetically divided into group 1 (H1, H2, H5, H6, H8, H9, H11, H12, H13, H16, H17, H18) and group 2 (H3, H4, H7, H10, H14, H15)^2^. The HA on the virion is in the prefusion state and cleaved by host proteases into HA1 and HA2^3^. In contrast to the highly variable antigenic region in the HA1 head domain, the HA2 stalk region is relatively conserved among the same HA group viruses, as recognized by broadly neutralizing antibodies among the different subtype viruses, supporting the HA2 stalk domain as a promising target for developing a universal vaccine^4,5^.

Previous studies reported that headless H1 stalk stabilized protein nanoparticle vaccines could provide protection against homologous H1N1^6,7^ and heterosubtypic H5N1 virus^6,8^. Group 2 HA stalk proteins were reported to be more challenging in stabilizing the trimers since additional modifications had to be introduced^9^. Headless H3 and H7 stalk protein vaccines were constructed and shown to be immunogenic, inducing protection against homologous H3N2 and H7N9 viruses respectively^9,10^. The efficacy of stalk-based particularly group 2 stalk vaccines was low with homo and heterologous viruses as evidenced by substantial body weight loss in mice and the breadth of viruses tested was very limited^6,9,10^. In addition, challenges exist in H3N2 subtypes compared to H1N1 subtypes because of stalk mutations in circulating strains and low fitness genetic barrier for H3N2 viruses in vitro and in vivo^11^. These drawbacks have been difficult challenges to overcome in developing effective H3 stalk-based vaccines.

Another promising antigenic target is the highly conserved extracellular domain of matrix 2 (M2e) protein in influenza A viruses^12,13^. M2e-based vaccines could provide broad cross protection against different strains and subtypes in mice^12–15^. Recombinant M2e vaccines were safe in phase 1 trials^13,16,17^. Low efficacy of M2e-based vaccines inducing non-neutralizing immunity is a concern for advancing toward a stand-alone vaccine. H1 plus H3 stalk protein vaccines layered onto the M2e core nanoparticles via chemical cross linking were reported to induce enhanced cross protection, compared to stalk or M2e alone vaccines^18^.

In this study, we constructed a chimeric M2e and H3 stalk vaccine by genetically linking M2e repeat to the engineered H3 stalk domain with stabilizing HA1 N- and C-terminal region and point mutations (M2e-H3 stalk). *E. Coli* expressed M2e-H3 stalk protein displayed multi conserved M2e and stalk epitopes that are recognized by antisera of both group 1 and 2 influenza virus infections and different subtype HA proteins. Adjuvanted M2e-H3 stalk protein vaccination induced broad protection against cross-group heterologous and heterosubtypic viruses despite a wider range of antigenic differences in adult and aged mice.

## Results

### Rationale design and development of chimeric M2e-H3 stalk universal vaccine construct

**Fig. 1:**
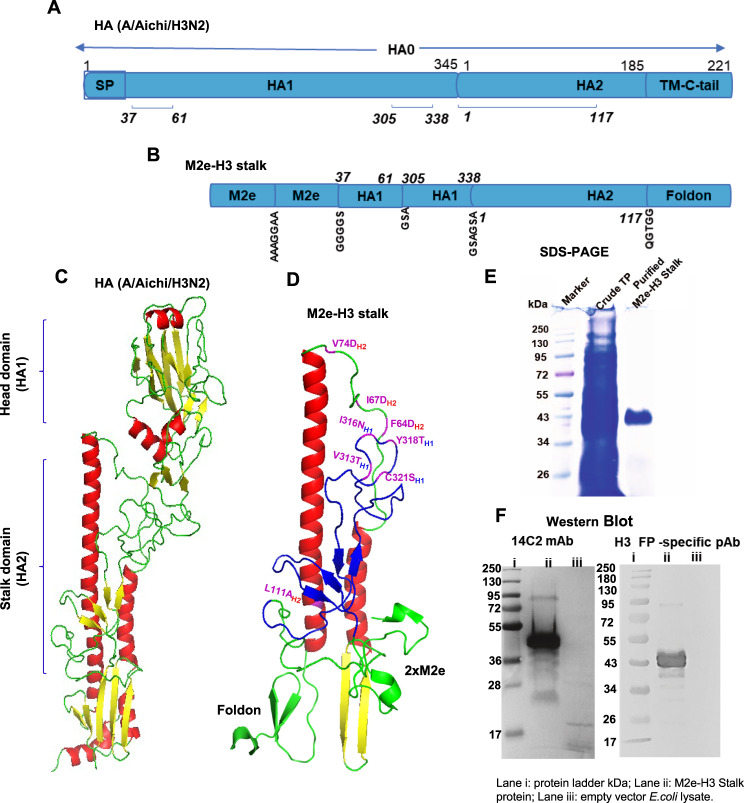
Rationale design of chimeric M2e-H3 stalk protein, purification, and confirmation. **A** Schematic of full-length HA gene of influenza A virus (A/Aichi/H3N2), and the selective domains as a vaccine target are numbered in amino acid (aa 37-61, 305-338, 1-117) residues. **B** M2e-H3 stalk vaccine construct with flexible and soluble linker sequences (AAAGGAA; GGGGS; GSA; GSAGSA; QGTGG). **C** The monomeric H3 HA 3D cartoon structure as predicted by the SWISS model and visualized in PyMol. **D** Illustration of monomeric cartoon structure of M2e-H3 stalk domain marking the positions of point mutations. M2e and foldon structures were modeled using PDB ID codes 4N8C and 1RFO, respectively. **E** Coomassie Blue staining of M2e-H3 stalk protein. Marker: protein size marker (kDa), Crude TP: Total cell lysates (25 µg); M2e-H3 stalk: purified M2e-H3 stalk protein (15 µg). **F** Western blot of M2e-H3 stalk protein. 14C2: M2e-specific mAb; stalk: anti-fusion peptide (FP) polyclonal antibody (pAb) recognizing HA2 aa1-14 epitope. **E**, **F** The original un-cropped images of all blots including full molecular weight markers are provided in the supplementary information file (Supplementary Figure S11).

Structural conformation of HA2 stalk domain was previously modeled to be stabilized with the N- and C-terminal HA1 parts^6,8^. To extend and enhance the breadth of cross protection, a genetic fusion of M2e epitopes and H3 stalk was constructed (Fig. 1A–D). The H3 shortened stalk domain contains HA1 parts [aa 37-61, aa 305-338 of H3 HA from A/Aichi], and HA2 stalk in α-helix conformation [aa 1-117, Fig. 1B, D]. Tandem 2x repeat of M2e (23 aa) epitope domains was genetically fused to the H3 stalk N-terminus (M2e-H3 stalk) from A/Aichi/H3N2 influenza A virus.

The N-terminal half of the HA2 stalk domain is enriched with broadly neutralizing B cell epitopes as previously identified^19–21^. Therefore, the C terminal hydrophobic stalk part was excluded in the M2e-H3 stalk construct and replaced with the β-rich trimeric nature of the foldon sequence to enhance the stability and proper folding of the protein (Fig. 1D). Point mutations shown in Fig. 1D were introduced in the hydrophobic patches in the HA1 (V313T_H1_, I316N_H1_, and Y318T_H1_) and HA2 stalk domains (F64D_H2_, I67D_H2_, V74D_H2_, L111A_H2_). These point mutations were previously described to attenuate strong hydrophobic interactions and to avoid protein aggregations in neutral pH conformation, potentially improving the protein preparation in a soluble form^7,22^. In addition, cysteine residue on 321 position was replaced by serine residue (C321S) to prevent non-specific intermolecular disulfide formation^7^. A previous study demonstrated that the foldon trimer stabilizing domain was required for helical trimer formation and thermal stabilization, and for enabling resistance to proteolysis^7^. To facilitate protein purification, 6xHis tag was fused to the N-terminus of the M2e-H3 stalk domain^23^. Flexible linkers were used to connect independent domains and to facilitate the display of native-like conformation.

A codon-optimized gene encoding M2e-H3 stalk protein was synthesized and cloned into pCold II, a high expression vector in *E. Coli*. Chimeric M2e-H3 stalk proteins were expressed in *E. Coli* cells. Cell lysates containing M2e-H3 stalk proteins were dissolved in 8M urea and fractions collected through the Ni-affinity His trap column were refolded into soluble M2e-H3 stalk protein with high purity (Fig. 1E). Chimeric M2e-H3 stalk proteins were further confirmed by western blot with M2e-specific mAb 14C2 and fusion epitope specific polyclonal antibody (pAb, Fig. 1F).

### Chimeric M2e-H3 stalk protein displays cross reactive antigenicity and thermostability

**Fig. 2:**
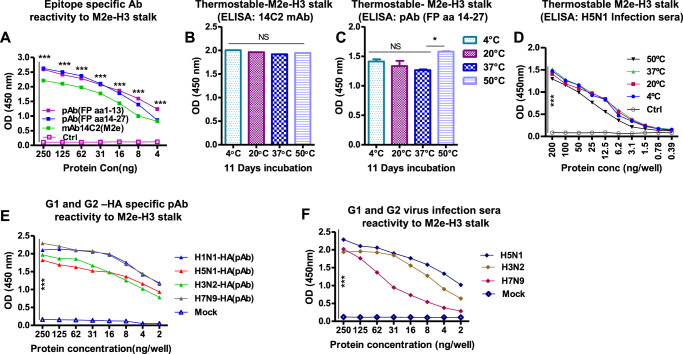
Characterization of M2e-H3 stalk protein antigenicity, stability, and its cross-reactivity. **A**, **E**, and **F** The antigenicity of purified M2e-H3 stalk protein was determined by standard ELISA using antibodies specific for M2e (14C2), HA2 domain (rabbit poly IgG Abs) purified against HA2 epitopes including aa1-13 (poly HA2#1–13) or aa14-27 (poly HA2#14-27), and polyclonal antibodies (pAbs) against recombinant HA proteins from different subtypes (H1N1, H5N1, H3N2, H7N9); antisera of mice infected with influenza A live viruses (rgH5N1, H3N2, rgH7N9) were used. **B**–**D** Thermostability of M2e-H3 stalk protein was evaluated after storage at different temperatures (4, 20, 37, 50 °C) for 11 days by determination of retaining antigenicity. **A** Antigen reactivity specific to conserved HA2 FP and M2e antibodies. **B** Thermostable M2e-H3 stalk protein reactivity to 14C2 mAb. **C** Thermostable M2e-H3 stalk protein reactivity to FP pAb. **D** Thermostable M2e-H3 stalk protein reactivity to H5N1 virus antisera. **E** M2e-H3 stalk protein reactivity to pAbs against HA proteins from group 1(G1) viruses (H1N1, H5N1) and group 2 (G2) viruses (H3N2, H7N9). **F** M2e-H3 stalk protein reactivity against the antisera of live G1 and G2 influenza A viruses. Ctrl: BSA control. Mock: Naïve mice sera. Statistical significance was determined by using one-way ANOVA followed by Tukey’s Multiple Comparison Test or two-way ANOVA followed by Bonferroni post-test; error bars indicate mean ± SEM; *P < 0.05;**P < 0.01; ***P < 0.001.

Epitope integrity and thermostability of chimeric M2e-H3 stalk protein were examined. M2e-H3 stalk proteins were highly reactive with M2e-specific mAb 14C2 as well as rabbit polyclonal antibodies specific for highly conserved HA2 aa1-13 fusion peptide, and HA2 aa14-27 peptide (Fig. 2A). M2e and fusion epitope antigenicity was retained even after incubation for 11 days at low (4 °C) to high temperature (50 °C) storage (Fig. 2B, C). The antigen was also reactive to antisera from mice recovered from H5N1 virus infection (Fig. 2D), indicating high thermostability of native-like epitope integrity. M2e-H3 stalk protein displayed strong antigenic reactivities for pAbs against HA proteins derived from diverse subtypes, including H1N1 (A/California/2009), H5N1 (A/Vietnam/2004), H3N2 (A/Swine/2011), and H7N9 (A/Shanghai/2013) (Fig. 2E). More importantly, antisera from infection with different subtype influenza A viruses (H5N1, H3N2, H7N9) exhibited high reactivities to the M2e-H3 stalk protein antigen (Fig. 2F). Overall, these results suggest that M2e-H3 stalk protein exposes diverse native-like conserved epitopes which are recognized by 14C2 mAb, different subtypes HA pAbs and antisera from virus infection.

### Adjuvanted M2e-H3 stalk protein vaccination induces IgG antibodies recognizing M2e, stalk, and diverse subtype viruses

**Fig. 3:**
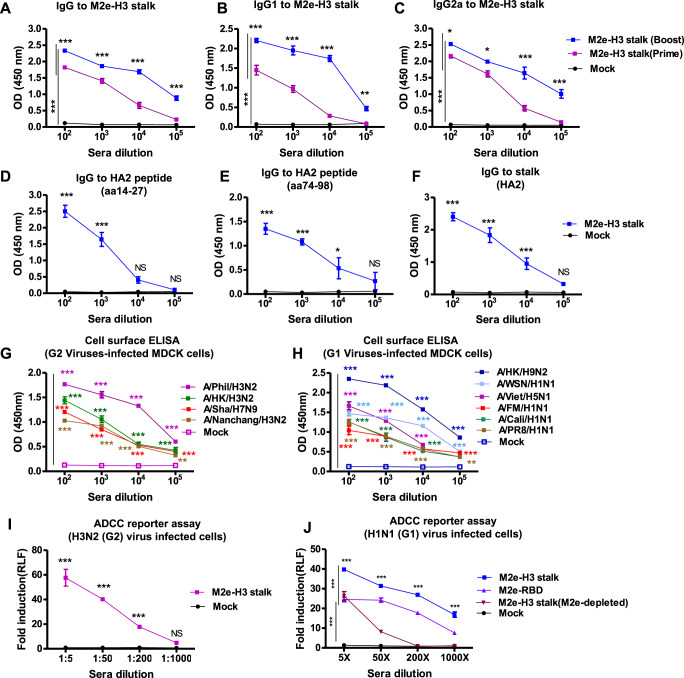
Adjuvanted M2e-H3 stalk vaccination induces antibodies recognizing M2e, FP, stalk, and group 1 and 2 viral antigens and virus-infected cell surface. BALB/c mice (*n* = 10) were intramuscular (i.m.) prime-boost immunized with adjuvanted M2e-H3 stalk (20 µg) protein. IgG antibodies and ADCC were determined in sera collected 2 weeks after vaccination. **A**–**C** IgG and IgG antibody subtypes specific for M2e-H3 Stalk protein. IgG (**A**), IgG1 (**B**) and IgG2a (**C**) antibodies specific for M2e-H3 stalk protein. **D**–**F** stalk-specific IgG antibodies in boost sera. IgG antibodies specific for HA2 FP epitope region aa14-27 of HA2 (**D**), stalk epitope residues aa74-98 of HA2 (**E**), and full-length stalk protein (aa 1-185 of HA2) (**F**). **G**, **H** M2e-H3 stalk vaccination induced IgG antibodies recognizing both group 2 (**G**) and group 1 (**H**) viral antigens on the surface of virus-infected MDCK cells. **I**, **J** Antibodies induced by M2e-H3 stalk vaccine engage in Fc-mediated activation of Jurkat effector cells, mimicking a surrogate ADCC activation pathway. Group 2 virus (G2) A/Phil/H3N2(H) and group 1 virus (G1) A/WSN/H1N1 (**J**). Mock: adjuvanted naïve sera, M2e-depleted: M2e-specific IgG depleted M2e-H3 stalk sera. Statistical significance was determined by using two-way ANOVA; error bars indicate mean ± SEM; *P < 0.05;**P < 0.01; ***P < 0.001.

Protein subunit vaccines in general require adjuvanted formulations to enhance the immune responses and protective efficacy. We tested the effects of AS01-like adjuvant (QS-21 + MPL), major ingredients in AS01 liposome adjuvant licensed for use in herpes Zoster vaccination^24,25^. Adjuvant (QS-21 + MPL) included in M2e-H3 stalk protein prime boost IM vaccination of mice with a 3-week interval exhibited significant impacts on enhancing IgG1 and IgG2a antibodies specific for M2e and M2e-H3 stalk antigens (Supplementary Fig. S1A–G). The impact on eliciting immune response with vaccine dosage and adjuvants was evaluated with different vaccine antigen amounts with or without adjuvants. The adjuvanted M2e-H3 stalk (20 µg) induced significantly higher IgG, IgG1, and IgG2 specific M2e-H3 stalk antibodies than the unadjuvanted group (Supplementary Fig. S1A–C). Four-fold less (5 µg) vaccine dose with adjuvanted M2e-H3 stalk induced higher levels of IgG responses than those with 20 µg of vaccination without adjuvant. Therefore, we focused on testing immune responses and efficacy after vaccination with adjuvanted (QS-21+MPL) M2e-H3 stalk vaccination of mice. At 2 weeks after M2e-H3 stalk protein (20 µg) prime vaccination, substantial levels of IgG, IgG1, and IgG2a specific to M2e-H3 stalk protein antigens were induced after IM prime dose (Fig. 3A–C). After boost, the levels of IgG, IgG1, and IgG2a antibodies specific to vaccine antigens or M2e epitope, were increased by approximately 10 folds (Fig. 3A–C and Supplementary Fig. S1D–G). Boost immune sera showed substantial levels of IgG antibodies binding to HA2 fusion peptides (aa14-27), HA2 stalk epitope (HA2aa 74-98), one of the essential epitopes on stalk domain, and stalk protein (Fig. 3D–F). Also, M2e-H3 stalk boost sera displayed higher levels of IgG antibodies against inactivated group 2 viruses (A/HK/1968/H3N2 and A/Phil/H3N2) than those inactivated group 1 viruses (rgA/HK/1999/H9N2 and A/California/2009/H1N1) (Supplementary Fig. S2A–D).

Furthermore, M2e-H3 stalk antisera strongly recognized both group 1 (G1) and group 2 (G2) viral antigens expressed on the surface of MDCK cells infected with a broad range of influenza A viruses (Fig. 3G, H). Antisera of adjuvanted M2e-H3 stalk vaccination were highly reactive to the cell surface viral antigens after infection with group 2 influenza A viruses, including A/Phil H3N2, A/HK H3N2, rgA/Shanghai H7N9, and A/Nanchang/1995 H3N2 (Fig. 3G). Interestingly, antisera of M2e-H3 stalk vaccination were also highly reactive to the cell surface viral antigens after infection with group 1 influenza viruses (A/Cal H1N1, rgA/HK H9N2, A/WSN/1933 H1N1, A/PR8 H1N1, A/FM H1N1, and A/Viet/H5N1) (Fig. 3H).

To investigate whether the M2e-H3 stalk vaccination would engage in Fc-mediated activation of effector Jurkat cells, supporting a possible role of antibody-dependent cellular cytotoxicity (ADCC) in providing cross-protection, we performed an ADCC assay. The ADCC surrogate reporter assay showed an elevated reporter signal with M2e-H3 stalk antisera on both group 2 A/Phil H3N2 (Fig. 3I) and group 1 (A/WSN/H1N1 in Fig. 3J, A/HK/H9N2 in Supplementary Fig. S8) influenza A virus-infected MDCK cells. In addition, M2e antibody-depleted M2e-H3 stalk antisera lost a significant fraction of their ADCC activity against A/WSN/H1N1 whereas M2e-RBD vaccine sera as a control of M2e alone antisera retained a substantial level of ADCC activity against A/WSN/H1N1 and showed less reduction in ADCC activity, as compared to the M2e-H3 stalk vaccine antisera (Fig. 3J). These ADCC experimental data suggest that M2e-specific IgG antisera might have played a significant role in exhibiting elevated levels of ADCC activity compared to the stalk antisera only when tested against A/WSN/H1N1 group 1 virus, and that the combination of M2e with H3 stalk antisera resulted in increasing the ADCC activity by over 20 folds. These results suggest that M2e-H3 stalk antisera has ADCC reporter assay activity against group 1 and group 2 influenza A viruses and that adjuvanted M2e-H3 stalk vaccination effectively induced antibodies recognizing M2e, stalk, and group 1 and 2 virus antigens on virions and on the surfaces of infected cells.

### M2e-H3 stalk protein vaccine provides broad and effective cross protection against group 2 viruses

**Fig. 4:**
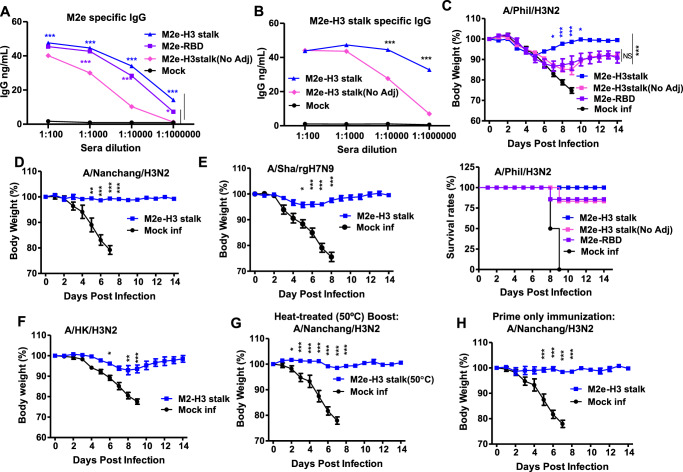
Adjuvanted M2e-H3 stalk vaccination induces heterologous cross-protection against group 2 influenza A viruses. **A** M2e-specific serum IgG antibody levels in the different vaccine groups. **B** Serum IgG antibodies specific for M2e-H3 stalk antigen in the M2e-H3 stalk groups with (M2e-H3 stalk) and without adjuvant (M2e-H3 stalk/No adj). **C**–**H** The groups of mice (*n* = 5, 6–8 weeks old) vaccinated (prime or prime-boost) with M2e-H3 stalk protein (20 μg +/- adjuvant) or M2e-RBD (20 μg plus adjuvant) were intranasally challenged with group 2 influenza A viruses (H3N2, H7N9). Body weight changes and survival rates were daily monitored. **C**, **D** A/Phil/1982 H3N2 (3xLD_50_, 2.3 × 10^2^ EID_50_), **E** A/Nanchang/1995 H3N2 (2xLD_50_, 3 × 10^6^ EID_50_), **F** A/Sha/2013 H7N9 (3xLD_50_, 1.1 × 10^4^ EID_50_), **G** A/HK/1968 H3N2 (3xLD_50_, 4 × 10^1^ EID_50_). **H** Efficacy of thermostable M2e-H3 stalk protein. Mice vaccinated with M2e-H3 stalk protein pre-incubated at 50 °C for 11 days prior to prime-boost vaccination were challenged with A/Nanchang/1995 H3N2 (2xLD_50_, 3 × 10^6^ EID_50_). **I** Efficacy of single dose M2e-H3 stalk vaccination. Mice with M2e-H3 stalk prime dose only were challenged with A/Nanchang/1995 H3N2 (2xLD_50_, 3 × 10^6^ EID_50_) at 4 weeks after vaccination. Mock inf: mock group (adjuvant only) with virus infection, No Adj: M2e-H3 stalk vaccinated group without adjuvant. Statistical significance was determined using the two-way ANOVA followed by Bonferroni post-test. Error bars indicate means ± SEM; *P < 0.05; **P < 0.01; ***P < 0.001.

Unadjuvanted M2e-H3 stalk protein (20 µg) vaccination resulted in weight loss up to 20% and survival rates 80% after challenge with A/Phil (H3N2) virus whereas unvaccinated mice did not survive A/Phil virus infection (Fig. 4A). The adjuvanted M2e-H3 stalk group exhibited significant higher efficacy of protection against lethal A/Phil virus infection, as evidenced by less weight loss (~8.5%) and 100% survival rates (Fig. 4A), supporting significant effects of adjuvant (QS-21+MPL) on enhancing the protection. To compare with the protection efficacy by M2e alone, we designed and purified another chimera protein (M2e-RBD) containing N-terminal M2e fused with receptor-binding domain (RBD) of SARS-COV-2 spike protein and stabilized with a foldon trimer (Supplementary Fig. S4). M2e-RBD protein was reactive with M2e-specific mAb (14C2, Supplementary Fig. S4C) and the adjuvanted M2e-RBD group induced high levels of M2e-specific antibodies (Supplementary Fig. S4D). The M2e specific and M2e-H3 stalk-specific IgG levels in the adjuvanted vaccine groups were significantly higher than unadjuvanted M2e-H3 stalk (Fig. 4A, B). However, the adjuvanted M2e-RBD group showed partial protection against A/Phil/H3N2 virus lethal challenge (Fig. 4C). Therefore, we extended the efficacy testing of adjuvanted M2e-H3 stalk vaccination to additional group 2 viruses. The adjuvanted M2e-H3 stalk group provided significantly enhanced protection against H3N2 (A/Nanchang/1995, Fig. 4D) and rgH7N9 (A/Shanghai/2013, Fig. 4E), as shown by minimum weight loss (<4%) and 100% survival rates (Supplementary Figs. S9A and S9B). A moderate level of weight loss (~9 %) was observed in the adjuvanted M2e-H3 stalk group after challenge with A/HK/1968 H3N2 virus (Fig. 4F and Supplementary Fig. S9C). These results indicate that adjuvanted M2e-H3 stalk protein vaccination provides effective cross protection against the antigenically different group 2 viruses.

Next, to evaluate the thermostability of the vaccine antigen, the M2e-H3 stalk was stored at high temperature (50 °C) for 11 days prior to vaccination. The vaccinated mice with M2e-H3 stalk after storage at 50 °C showed 100% protection against A/Nanchang/H3N2 virus challenge (Fig. 4G and Supplementary Fig. S9D). A single dose of M2e-H3 stalk (20 µg) vaccination showed 100% protection against the A/Nanchang/H3N2 virus, preventing weight loss, while the mock group did not survive (Fig. 4H and Supplementary Fig. S9E). These results revealed that M2e-H3 stalk protein is thermostable, and prime-only vaccination can provide protection against the group 2 influenza A viruses.

### M2e-H3 stalk protein vaccination prevents severe weight loss and confers cross protection against group 1 viruses

**Fig. 5:**
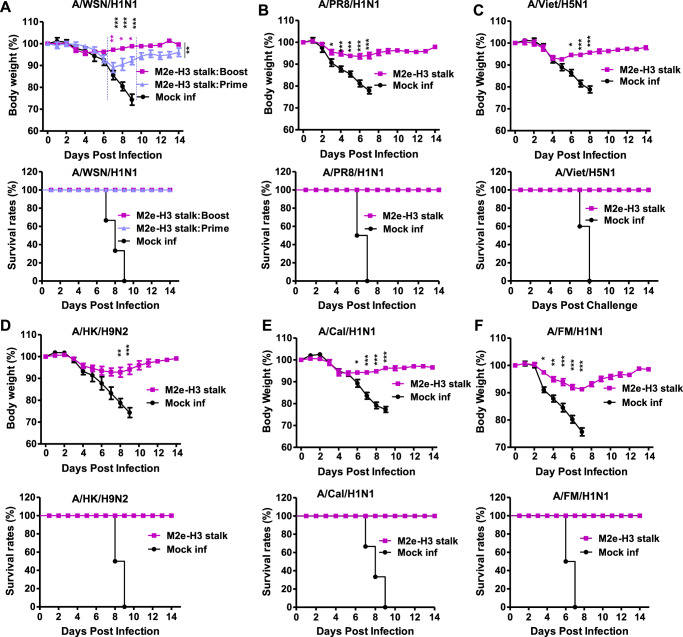
Adjuvanted M2e-H3 stalk protein provides protection against heterologous cross-group 1 influenza A viruses. The groups of mice (*n* = 5, 6–8 weeks old) vaccinated with adjuvanted M2e-H3 stalk (prime and prime-boost for WSN/H1N1 virus challenge group or prime-boost for the rest of virus challenge) were intranasally challenged with group 1 influenza A viruses (H1N1, H5N1, H9N2). Body weight changes and survival rates were monitored for 14 days. **A** A/WSN/1933 H1N1 (2xLD_50_, 1.5 × 10^2^ EID_50_), **B** A/PR8/1934 H1N1(4xLD_50,_1.2 × 10^3^ EID_50_), **C** A/Viet/2004 rgH5N1 (3xLD_50_, 2.6 × 10^4^ EID_50_), **D** A/HK/1999 H9N2 (4xLD_50_, 7.8 × 10^1^ EID_50_), **E** A/Cal/2009 H1N1(3xLD_50_, 2 × 10^3^ EID_50_), **F** A/FM/1947 H1N1(3xLD_50_, 8 × 10^3^ EID_50_). Mock inf: mock group (adjuvant only) with virus infection. Statistical significance was determined using the two-way ANOVA followed by Bonferroni post-test. Error bars indicate means ± SEM; *P < 0.05; **P < 0.01; ***P < 0.001.

HA2 stalk immunity is known to be group specific and ineffective in inducing cross group protection^6,9,10^. Here we tested whether adjuvanted M2e-H3 (group 2) stalk protein vaccination would induce protection against group 1 viruses after 4–8 weeks post boost dose. The M2e-H3 stalk primed group showed complete protection against group 1 virus (A/WSN), although the mice displayed moderate weight loss (~10%) whereas minimum weight loss (<4%) was observed in the M2e-H3 stalk boosted group (Fig. 5A). Prevention of severe weight loss as well as 100% survival rates were observed with adjuvanted M2e-H3 stalk protein vaccination after lethal challenge with H1N1 viruses (A/WSN/1933, A/PR8), H5N1 virus (rgA/Vietnam/2004), and H9N2 virus (rgA/HK/1997) (Fig. 5A–D). Cross-group protection against other additional H1N1 strains such as A/California/2009 and A/FM/1947 was observed with the outcomes of a low-level weight loss (5–8%) and 100% survival rates after adjuvanted M2e-H3 stalk protein vaccination of mice (Fig. 5E, F). These results support that adjuvanted M2e-H3 stalk protein vaccination can provide cross-group protection and prevent severe weight loss.

### M2e-H3 stalk protein vaccination induces effective lung viral clearance and protective humoral and cellular immune responses

**Fig. 6:**
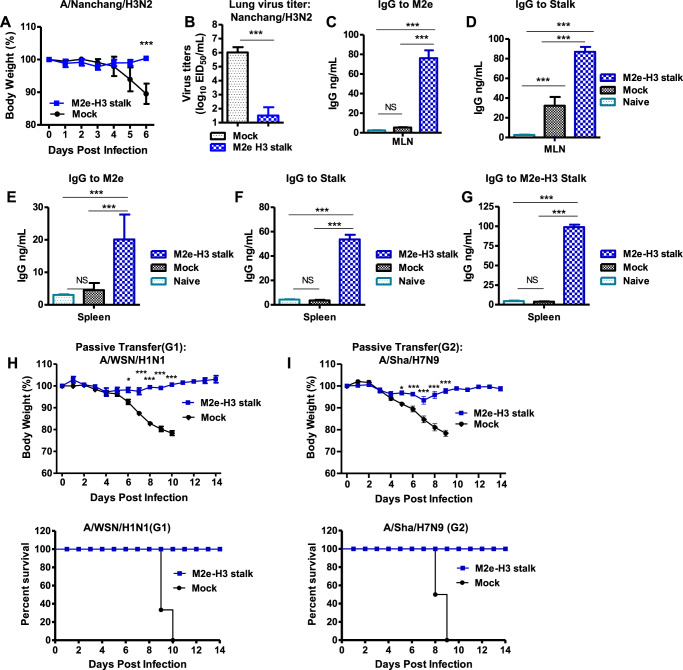
Adjuvanted M2e-H3 stalk vaccination bestows cross protection by lowering lung viral loads and inducing protective humoral immune responses. **A** Body weight changes in M2e-H3 stalk (20 µg) vaccinated young BALB/c mice (*n* = 5) for 6 days after challenge with lethal dose (2xLD_50_, 3 × 10^6^ EID_50_) of A/Nanchang/H3N2 virus. **B** Lung viral titers at 6 days post-infection. **C**–**G** In vitro production of IgG antibodies specific for M2e (**C**) and stalk (**D**) in mediastinal lymph node (MLN) and spleen cultures IgG antibodies specific for M2e (**E**), stalk (**F**) or M2e-H3 stalk (**G**), determined by ELISA. **H**, **I** Protective efficacy of vaccine immune sera. Body weight changes in naïve mice after intranasal inoculation with a mixture of M2e-H3 stalk vaccine immune sera or naive sera and group 1 influenza A virus (A/WSN H1N1, 4.2xLD_50_) (**H**) and group 2 influenza A virus (A/rgH7N9, 5xLD_50_) (**I**). Mock: mock group (adjuvant only no vaccine group (**A**–**G**) with virus infection). Naïve: mice group with no immunization and no virus infection. Mock: adjuvanted naïve sera (**H**, **I**). Statistical significance was determined by using one-way ANOVA followed by Tukey’s Multiple Comparison Test or two-way ANOVA followed by Bonferroni post-test; error bars indicate mean ± SEM; *P < 0.05; **P < 0.01; ***P < 0.001.

For better assessment of protective efficacy, we analyzed lung viral titers, humoral and cellular immune responses. M2e-H3 stalk vaccination significantly reduced lung viral titers by a magnitude of over 4 log10 day 6 post-challenge with A/Nanchang/1995(H3N2) compared to mock group (Fig. 6B). M2e and stalk-specific IgG secreting cell responses were determined in the culture supernatants of MLN and spleens collected at day 6 after challenge with rgA/Nanchang/1995 (H3N2) (Fig. 6C–G). M2e-specific IgG antibodies were secreted in both MLN and spleen cell cultures only from M2e-H3 stalk vaccinated mice (Fig. 6C, E). Also, significantly higher levels of stalk-specific IgG antibodies were produced in both MLN and spleen cell in vitro cultures from M2e-H3 stalk vaccinated mice (Fig. 6D, F).

To evaluate the role of humoral immune responses in providing cross-protection, naïve mice were intranasally infected with a mixture of lethal dose virus and boost antisera of adjuvanted M2e-H3 stalk vaccination (Fig. 6H–I). Consistent with cross group protection, the naïve mice inoculated with a mixture of group 1 virus (A/WSN/H1N1) and M2e-H3 stalk boost sera showed complete protection without severe weight loss. In contrast, the control mice given this mixture of naïve sera and virus did not survive (Fig. 6H). Also, the naïve mice infected with lethal group 2 virus (rgA/H7N9) and M2-H3 stalk antisera were protected, despite a moderate weight loss (<10%), whereas mock sera with rgA/H7N9 virus failed to provide any protection (Fig. 6I).

**Fig. 7:**
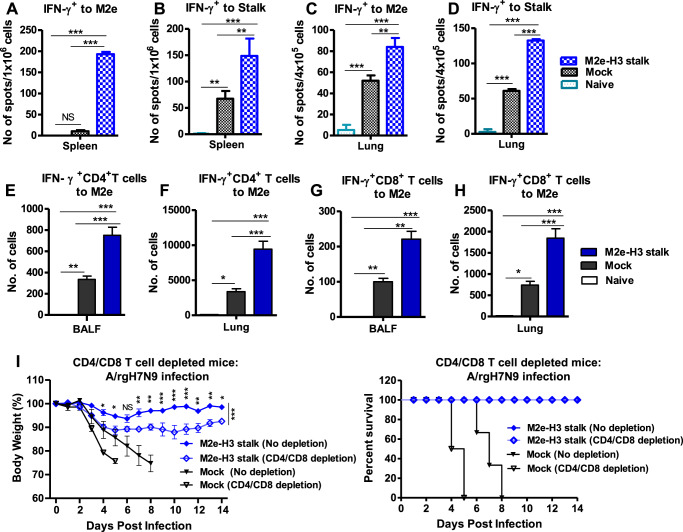
Adjuvanted M2e-H3 stalk vaccine induces protective T-cell immunity. Cytokine-secreting T-cell immune responses were evaluated in M2e-H3 stalk immunized mice at 6 days post-infection with A/Nanchang H3N2 virus. **A**–**D** IFN-γ+ secreting ELISpot assays of spleen cells after in vitro antigen stimulation with M2e- (**A**) or stalk (**B**), and in Lung cells stimulated with M2e (**C**) or Stalk (**D**). **E**–**H** IFN-γ^+^ CD4^+^ or CD8^+^ T cells responses in BALF and Lung cells were determined by intracellular cytokine staining and flow cytometry analysis. IFN-γ^+^ CD4^+^ T cells response in BALF (**E**) and Lung (**F**). IFN-γ^+^ CD8^+^ T cells response in BALF (**G**) and Lung (**H**). Mock: mock group (adjuvant only) with virus infection. Naïve: mice group with no immunization and no virus infection. Impact of CD4^+^ and CD8^+^ T-cell depletion on protection in M2e-H3 stalk vaccinated mice before challenging with A/rgH7N9 virus (**I**). Statistical significance was determined by using one-way ANOVA followed by Tukey’s Multiple Comparison Test or two-way ANOVA followed by Bonferroni post-test; error bars indicate mean ± SEM; *P < 0.05; **P < 0.01; ***P < 0.001.

T-cell immune responses were assessed by ELIspot assay and flow cytometry analysis (Fig. 7A–H). M2e-stimulated IFN-γ^+^ secreting splenocyte cell spots were observed only in M2e-H3 stalk vaccinated mice at significant levels (Fig. 7A, C). Stalk-stimulated IFN-γ^+^ secreting splenocyte cell spots were induced at higher levels by adjuvanted M2e-H3 stalk vaccination than those in naïve mice as determined day 6 post infection (Fig. 7B, D). Intracellular cytokine staining and flow cytometry analysis indicated significantly enhanced levels of M2e-specific IFN-γ^+^ CD4^+^ T cells and IFN-γ^+^ CD8^+^ T cells in the airway bronchoalveolar lavage fluid (BALF) and lung samples in the adjuvanted M2e-H3 stalk group compared to those in naïve mice collected day 6 post infection (Figs. 7E–H and Supplementary Fig. S10). These data suggest that adjuvanted M2e-H3 stalk vaccination induces enhanced lung viral clearance and humoral and cellular immune responses.

In addition, the contribution of T-cell immunity to cross-protection was determined in boost-vaccinated M2e-H3 stalk by depleting CD4^+^ and CD8^+^ cells before lethal challenge with rgA/Shanghai (H7N9) virus (Fig. 7I). Depleting CD4 and CD8 T cells in M2e-H3 stalk vaccinated mice resulted in significantly more weight loss (~12%) than the T cell-nondepleted M2e-H3 stalk vaccinated mice which displayed minimum weight loss (~6%) and quickly recovered, supporting the contribution of T cells to enhancing cross-protection.

### M2e-H3 stalk vaccine provides effective protection against both group 1 and 2 viruses in old-aged mice

**Fig. 8:**
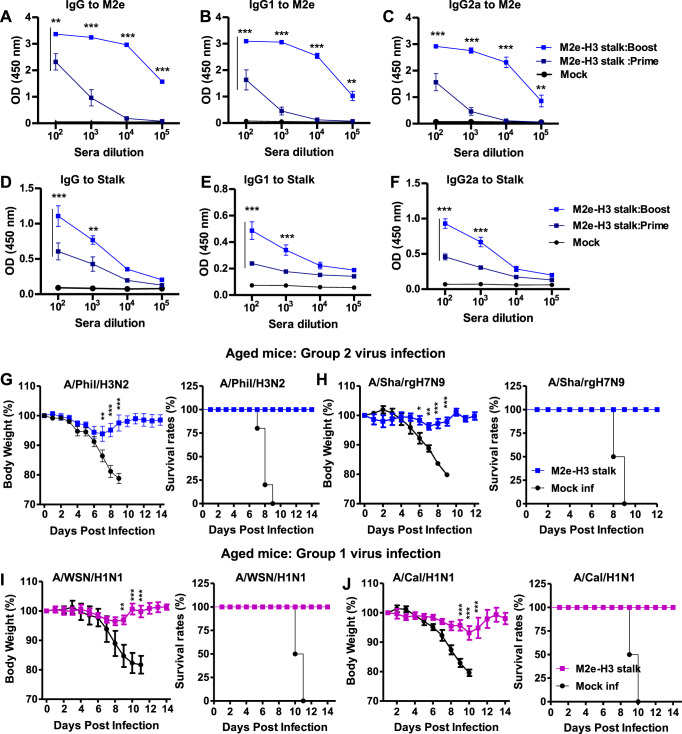
Aged mice with adjuvanted M2e-H3 stalk vaccination induces cross-group virus protection. Aged mice (16 months old, *n* = 5) were i.m. prime-boost immunized with adjuvanted M2e-H3 stalk protein. **A**–**F** M2e-H3 stalk vaccination of aged mice induced IgG, IgG1 and IgG2a antibodies against M2e and stalk protein. IgG to M2e (**A**), IgG1 to M2e (**B**), IgG 2a to M2e (**C**). IgG to stalk (**D**), IgG1 to stalk (**E**), IgG 2a to stalk (**F**). **G**–**J** Efficacy of cross-group virus protection in aged mice as measured by body weight changes and survival rates. Aged mice (*n* = 5) at 3 weeks after boost vaccination were intranasally challenged with a lethal dose of either group 1 (A/WSN H1N1, A/Cal H1N1) or group 2 (A/Phil H3N2, A/Sha/ H7N9) influenza A viruses. A/Phil H3N2 (**G**), A/Sha/ H7N9 (**H**), A/WSN H1N1 (**I**), A/Cal H1N1 (**J**). Statistical significance was determined using the two-way ANOVA followed by Bonferroni post-test. Error bars indicate means ± SEM; *P < 0.05; **P < 0.01; ***P < 0.001.

Groups of old aged (16 months old) mice were prime-boost vaccinated with adjuvanted M2e-H3 stalk protein (20 µg) at a 3-week interval. High levels of M2e (Fig. 8A–C) and stalk-specific IgG antibodies were induced by adjuvanted M2e-H3 stalk vaccination of aged mice (Fig. 8D–F). Similarly, the levels of IgG specific for M2e-H3 stalk were significantly increased after boost in aged mice (Supplementary Fig. S3A–C). The vaccinated aged mice were protected against A/Phil (H3N2, group 2) virus challenge at 8 weeks after boost and prevented weight loss to minimum (<5%) compared to the mock control group displaying severe weight loss (>20%) with partial 50% survival rates (Fig. 8G). Consistent, high efficacy of protection against a lethal dose of rgA/Shanghai/2013 (H7N9, group 2) was observed with minimum weight loss (<5%) in the vaccinated aged mice (Fig. 8H). Similarly, adjuvanted M2e-H3 stalk vaccination of aged mice provided protection against H1N1 group 1 viruses (A/California/2009, A/WSN/1933), preventing severe weight loss (<7%) whereas the mock control mice did not survive H1N1 virus infection (Fig. 8I, J). These results suggest that M2e-H3 stalk vaccination induces effective protection against both group 1 and 2 viruses in old-aged mice.

### M2e-H3 stalk protein vaccine dosage effects on inducing IgG responses and protection efficacy with M2e only vaccines

We tested different dosage effects of M2e-H3 stalk protein (5 µg, 10 µg, 20 µg) on inducing IgG responses and protection after prime boost adjuvanted vaccination. There were no significant differences in the levels of IgG Abs specific for M2e and stalk antigens between the 10 µg and 20 µg vaccine dose groups whereas the 5 µg dose group induced lower levels of M2e and M2e-H3 stalk binding antibodies than those in the higher dose groups (Supplementary Fig. S1A–E).

**Fig. 9:**
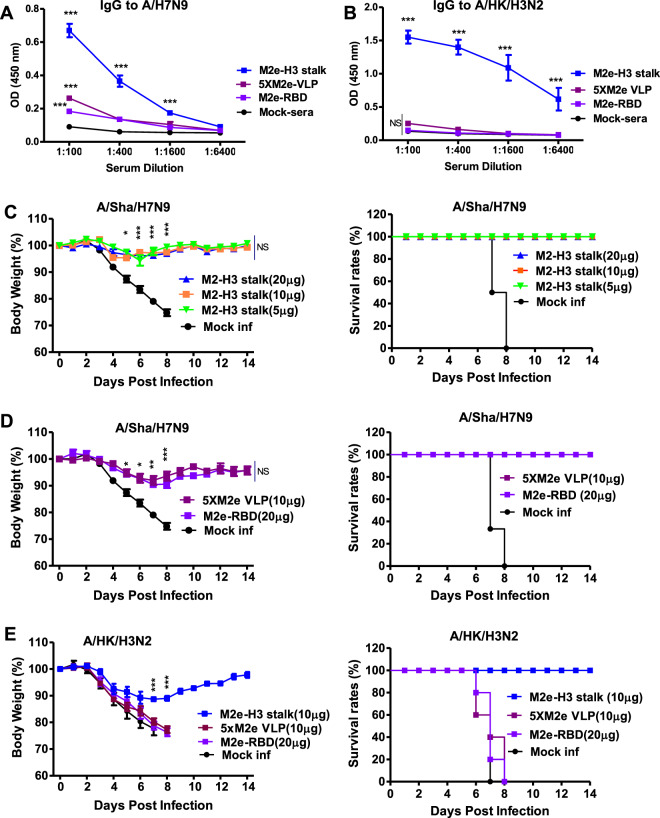
Adjuvanted M2e-H3 stalk vaccine dosage effects and protective advantages over M2e only vaccines. Young adult mice (6–8 weeks old, *n* = 5) were vaccinated with 5xM2e VLP (10 µg) or M2e-RBD (20 µg) or M2-H3 stalk protein at different doses (5 µg,10 µg, 20 µg). **A** IgG antibodies specific for A/Sha H7N9 virus. **B** IgG antibodies specific for A/HK H3N2 virus. **C** Vaccine dosage effects of M2e-H3 stalk on body weight changes and survival rates after A/Sha/2013 H7N9 (3xLD_50_, 1.1 × 10^4^ EID_50_) virus challenge. **D** Protective efficacy of M2e only vaccines after A/Sha/2013 H7N9 (3xLD_50_, 1.1 × 10^4^ EID_50_) virus challenge. **E** Protective efficacy comparison of M2e-H3 stalk and M2e only vaccines after A/HK/1968 H3N2 (12xLD_50_, 1.4 × 10^2^ EID_50_) virus challenge. Statistical significance was determined using the two-way ANOVA followed by Bonferroni post-test. Error bars indicate means ± SEM; *P < 0.05; **P < 0.01; ***P < 0.001.

We also compared M2-H3 stalk vaccine immunogenicity and efficacy with M2e only vaccines (M2e-RBD, 5xM2e VLP). High levels of IgG antibodies specific for M2e were induced by vaccination with M2e-H3 stalk, M2e-RBD, or 5xM2e virus-like particle (VLP) without significant differences among the groups (Supplementary Fig. S5A). The group of M2e-H3 stalk but not M2e only vaccination induced IgG antibodies highly reactive to inactivated group 2 viruses such as A/H7N9 and A/HK/H3N2 (Fig. 9A, B). In addition, M2e-H3 stalk vaccination induced significantly higher levels of IgG antibodies binding to cell-expressed viral antigens after infection of MDCK cells with group 2 viruses (A/Nanchang/H3N2, A/HK/H3N2) and group 1 viruses (A/WSN/H1N1, A/H9N2) than those by M2e alone vaccination (Supplementary Fig. S5B–E).

The groups of mice vaccinated with different doses (5 ug, 10 ug, 20 ug) of M2e-H3 stalk were similarly well protected against rgA/Shanghai/H7N9 virus, preventing weight loss (Fig. 9C). Meanwhile, the groups of mice vaccinated with 5xM2e VLP (10 ug) or M2-RBD (20 ug) displayed a moderate level (~10%) weight loss after rgA/Shanghai/H7N9 virus challenge (Fig. 9D). The mock group did not survive after virus infection. After challenge with a high lethal dose of A/HK/H3N2 virus (10x LD_50_), all mice in the 5xM2e VLP group died of infection (Fig. 9E). The M2e-H3 stalk group was completely protected against A/HK/H3N2 virus challenge, displaying only a moderate level (10%) weight loss. The mediastinal lymph nodes (MLN) collected from either the M2e-H3 stalk or M2e-RBD vaccination group, after challenging with A/Nanchang/H3N2 virus, were highly effective in inducing rapid plasma cell responses secreting M2e-specific IgG antibodies (Supplementary Fig. S6A). As expected, only the M2e-H3 stalk group showed IgG antibodies specific for stalk domain at significantly higher levels in culture supernatants of MLN (Supplementary Fig. S6B). These results support that chimeric M2e-H3 stalk vaccine can be more effective in inducing cross protection than M2e alone based vaccine construct.

## Discussion

Group 1 stalk only vaccines were ineffective in inducing cross protection against heterosubtypic and group 2 viruses due to sequence variations^26,27^. In particular, the vaccine effectiveness against H3N2 over the past decade was in a low range of approximately 33%^28^ and significantly low down to 6% during the 2014–2015 season^1^. New H3N2 variants with drifting mutations emerged with increased virulence^29^. The outbreak of group 2 H7N9 subtype virus represented one of potential pandemics^30^. Thus, it is of high priority to develop an effective group 2 or cross group vaccine. Here we presented a unique design and successful expression of M2e-H3 stalk protein construct in *E. coli*, which was thermostable and antigenically exposing conserved M2e, fusion peptide, and native-like stalk epitopes recognized by antisera of live group 1 and 2 virus infections. Vaccination of mice using M2e-H3 stalk protein with adjuvant (QS-21 + MPL) induced IgG antibodies specific for M2e, HA stalk, and group 2 viruses and, to a lesser degree, group 1 viruses. Mice with adjuvanted M2e-H3 stalk vaccination were broadly protected against both group 1 and 2 viruses, supporting further development as a promising universal influenza A vaccine candidate.

Earlier studies reported isolation and characterization of human monoclonal antibodies (mAbs: FI6v3, CR9114, CT149) that could broadly neutralize group 1 and 2 viruses^20,31,32^. Footprint and X-ray crystallography studies revealed the contact area between broadly neutralizing mAbs and HA conserved domains and residues. The group 1 HA stem mAbs (C179, CR6261, F10) were shown to contact the HA2 stem N-terminal fusion peptide region and helix A domain proximal to the membrane as well as the N- and C-terminal regions of HA1, with some differences among the mAbs^5,21,33,34^. The contact sites of group 2 HA stem mAbs (CR8020, CR8043) and cross-group mAbs (FI6v3, CR9114, CT149) were mapped to be HA2 multi-domains including the fusion peptide C-terminal region, an outmost edge of the β-sheet and helix A (Fig. 1D)^5^. Although the contact sites were overlapping with those of group 1 mAbs, the group 2 and cross-group mAbs appeared to have some differences such as a larger area of contact spanning the fusion peptides, the viral membrane proximal outer β-strands preceding helix A (Fig. 1D). These epitope mapping data of broadly neutralizing mAbs have provided a rational design of stalk stabilized immunogens. Corbett et al. (2019) reported group 2 headless H3 stalk-stabilized (H3ssF) and H7 stalk-stabilized (H7ssF) ferritin nanoparticle immunogens constructed through helix stabilization (HA2 aa 60-92 replaced with a G-rich loop), B loop optimization, and multiple point mutations, and expressed in mammalian cells^9^. Vaccination of mice with adjuvanted H3ssF and H7ssF immunogens induced subtype virus specific neutralizing activities and moderate efficacy of protection against lethal challenge^9^. However, H3ssF and H7ssF immunogens did not induce heterologous cross neutralizing Abs and cross protection remains unknown, suggesting a limitation of stem only vaccination^9^. In an attempt to overcome limited cross protection, we designed a chimeric M2e-H3 stalk construct expressed in *E. coli*. The M2e-H3 stalk contains M2e repeat, HA1 stem-interacting fragments, HA2 aa1-117 stem domain composed of fusion peptide with membrane proximal β-strands, helix A, loop B with point mutations, and helix C, which covers most epitopes known for broadly neutralizing stalk mAbs (Fig. 1D). Antigenicity data of M2e-H3 stalk suggest the presentation of native-like conserved epitopes to be exposed for recognition by Abs specific for both group 1 and 2 HA and antisera from infection with live viruses in addition to M2e and stalk.

Full-length H3 stalk (aa 1-172) proteins with point mutations to retain prefusion-like conformation and headless HA1 fragments expressed in *E. coli* provided low efficacy of homologous and partial heterologous H3N2 protection in mice^22^. In a follow up study, *E. coli* expressed, reduced sizes of H3 stalk helix domains (aa 44-113) with similar mutations and HA1 fragments conferred only partial survival protection (40-50%) against homologous H3N2 virus despite the induction of cross-reactive IgG Abs^35^. Sutton et al. (2017) demonstrated that the group 2 headless H3 and H7 stalk immunogens with several residues extended (aa 37-115) and stabilizing point mutations, expressed in *E. coli*, induced homologous H3N2 protection with minimum weight loss but low efficacy of cross protection against heterologous H3N2 or heterosubtypic H7N9 virus as shown by severe weight loss and partial survival rates^10^. Although it is difficult to have direct comparisons to those in previous studies, the M2e repeat and fusion peptide regions with the membrane proximal outer β-strands, helix A and C in M2e-H3 stalk (HA2 aa 1-117, Fig. 1), which contain shared and conserved epitopes recognized the cross group broadly neutralizing mAbs (FI6v3, CR9114, CT149), might have contributed to broad and high cross group protection.

Stabilization of the group 2 HA headless-stalk proteins required additional modifications as compared to the group 1 HA stalk^9^. Here we demonstrate that M2e-H3 stalk protein vaccination induced broad cross-protection against both group 1 (H1N1, rgH5N1, rgH9N2) and group 2 (H3N2, rgH7N9) influenza A viruses, which supports that M2e-H3 stalk protein can be developed as a potential universal vaccine candidate. As expected, H3N2 virus specific IgG responses were induced at higher levels by M2e-H3 stalk vaccination than those for intragroup heterosubtypic (rgH7N9) or cross-group viruses (H1N1, rgH9N2). The HA stalk sequence homology is as high as over 94% among the same H3N2 subtype viruses but reduced to below 70% among different subtypes within the group 2, and further down to 60% homology with among the group 1 viruses (Supplementary Fig. S7). Despite the low homology of stalk sequences, M2e-H3 stalk provided cross protection against heterosubtypic rgH7N9 and group 1 viruses such as H1N1 (A/WSN, A/PR8, A/FM, A/Cal/2009), rgH5N1, and rgH9N2. The efficacy of cross protection by M2e-H3 stalk was significantly improved as evidenced by preventing weight loss in mice under lethal challenge. Particularly, it is the first time to report that M2e-H3 stalk provided cross protection against both group 1 and 2 viruses in aged mice without apparent weight loss under lethal challenge. Since the mortality rates of seasonal influenza viruses are relatively low, a condition of lethal dose challenge is considered appropriate to assess the efficacy of cross protection. In contrast, previous studies reported severe weight loss after heterologous challenge in mice with adjuvanted headless-stalk vaccination^7,22,35^. Nonetheless, it is not possible to compare the cross protective efficacy with other studies, due to the differences in vaccine doses, adjuvants used, challenge virus and doses, and the number of vaccinations.

There are several possible immune mechanisms for broad cross-group virus protection by M2e-H3 stalk vaccination. M2e humoral and cellular immunity has been known to provide cross protection against both group 1 and 2 viruses^36^. M2 is expressed on the infected cell surfaces at high levels but incorporated into influenza virions at very low levels^37^. Fc receptors (FcR) were reported to play a critical role for cross protective immunity by M2e and stalk-based vaccination^38–40^, suggesting clearance of virus-infected cells or virion immune complexes via ADCC, antibody-dependent cellular phagocytosis, and complement-dependent cytolysis. In support of ADCC as a possible protective mechanism, M2e-H3 stalk immune sera exhibited high levels of binding IgG Abs to various group 1 and 2 viral antigens expressed on the cell surface as well as high activity of ADCC surrogate effector Jurkat cells on the MDCK cells infected with group 1 and 2 viruses. Passive transfer of antisera of M2e-H3 stalk conferred cross-protection against both group 1 (A/WSN, H1N1) and 2 (rgH7N9) viruses, supporting a significant role of humoral immunity. In addition, M2e is known to contain epitopes for T cells in mice and humans^13,41^. In support of T-cell immunity, we observed substantial levels of IFN-γ expressing cell spots and particularly IFN-γ producing CD4^+^ T cells in M2e-H3 stalk vaccinated mice. Also, depletion of T cells from M2e-H3 stalk vaccinated mice resulted in lower efficacy of protection, suggesting an important role of T cells in cross protection. Enhanced group specific cross protection was previously reported in mice with double-layer nanoparticle vaccine prepared by M2e cores chemically cross linked to H1 or H3 stalk proteins expressed in insect cells^18^. M2e-H3 stalk protein is unique in structural design, containing known epitopes of broadly neutralizing mAbs, thermostable, and in conferring cross protection against a wide range of cross group viruses including H1, H3, H5, H7, and H9 subtypes. Chimeric M2e-H3 stalk protein overcomes the limitation of M2e alone or stalk only based vaccines. *E. coli* bacterial expression of M2e-H3 stalk enables rapid scale-up during a pandemic outbreak even in low resource countries. Further studies will be needed for better mechanistic understanding and correlates of cross group protection and to test the efficacy of M2e-H3 stalk as a universal influenza vaccine candidate in a more relevant ferret animal model to support the clinical trials.

## Methods

### Rationale molecular design and synthesis of M2e-H3 stalk vaccine construct

HA gene sequence of influenza A virus [A/Aichi/12/1968 (H3N2)] was obtained from GenBank (ID: M55059) and used to design the H3 stalk vaccine construct. The conserved domains of HA were identified by multiple alignment of influenza A virus sequences. The amino acid (aa) residues of the HA1 (aa37-61 and 305-338) and HA2 (aa 1-117) domains were included as a vaccine target based on the major conserved region of the HA stalk and stabilizing domain. Point mutations were introduced on the hydrophobic aa residues of the targeted fragments by replacing with polar and hydrophilic residues without affecting the structure of the HA stem. Cysteine 321 was replaced with serine (C321S) on the HA1 region. The conserved M2e sequence (SLLTEVETPIRNEWGSRSNDSS) repeat was introduced in the N terminal and the foldon sequence was connected to the C terminal of the selected H3 stalk domain. The structure of M2e and foldon was derived from the protein data bank (PDB) ID codes 4N8C and 1RFO, respectively. The 3D structure of HA was predicted using the SWISS model and visualized in PyMol. The newly designed vaccine construct was named as M2e-H3 stalk. The nucleotide sequence of the M2e-H3 stalk construct was codon-optimized for expression in *Escherichia coli* (*E. Coli*) and synthesized by Genscript (USA). In addition, to develop an M2e alone based vaccine, receptor-binding domain (RBD) of SARS-CoV-2 was fused with the M2e epitope and β-rich trimeric nature of foldon sequence on the N and C terminal with soluble linker sequences, respectively. The vaccine construct was codon-optimized for *E. coli*; synthesized and named as M2e-RBD.

### Gene cloning, protein expression, and purification

The synthesized M2e-H3 stalk gene was ligated into the *NdeI* (5′ end) and *HindIII* (3′ end) restriction enzyme site within the pCold II cold expression vector (cat no:3362, Takara Bio. Inc) containing N-terminal 6x His-tagged, and subsequently transformed into *E.coli* Rosetta (DE3) pLysis cells (cat no: 709563, Novagen, USA). The protein expression was induced with 1 mM of isopropyl-β-d-thiogalactopyranoside for 12–14 h at 16 °C when the OD600 reached 0.4–0.5. The protein expressed cells were harvested by centrifugation and resuspended in lysis buffer containing 20 mM HEPES, pH 8.0, 300 mM NaCl, 2 mM CHAPS, 8 M urea, 10 mM imidazole, followed by sonication; cleared lysates were applied to His tag affinity Ni-NTA beads (Qiagen, USA). The beads were washed with lysis buffer containing 25 mM imidazole. The bound protein was eluted with a lysis buffer containing 250 mm imidazole. Purified protein was refolded by step dialysis in 20 mM HEPES, pH 8.0, 200 mM NaCl, 5% glycerol, and 1 mM DTT with a gradual decrease in the concentration of urea. The final refolded protein was further dialyzed using PBS (overnight at 4 °C), quantified, and stored at −80 °C until further use. Similarly, the M2e-RBD protein was purified and characterized. 5xM2e VLP (5xM2e tandem repeats) vaccine was prepared using the baculovirus system^14^, and used as one of the M2e-based vaccine controls. The purified proteins were separated by 12% sodium dodecyl sulfate-polyacrylamide gel electrophoresis (SDS-PAGE) and evaluated by western blot using M2e-specific 14C2 mAb and H3-stalk specific rabbit pAbs. The M2e and stalk epitopes in the purified M2e-H3 stalk protein were determined by standard ELISA using M2e and stalk epitope-specific antibodies and live virus antisera.

### Antibodies, peptides, and protein

M2e-specific mAb (14C2) was purchased from Santa Cruz Biotechnology (cat no: sc-32238, USA). The following rabbit polyclonal antibodies (pAbs) specific for stalk were generated by GenScript (USA): anti-fusion peptide (HA2aa1-13: GLFGAIAGFIEGG) and anti-H3-FP specific for HA2aa14-27 (WEGMVDGWYGFRHQ). Goat polyclonal antibodies (pAbs) specific for recombinant HA were acquired from BEI resources (ATCC/NIH): anti-H1 HA pAbs (NR 15696); anti-H5 HA pAbs (NR 2705); anti-H3 HA pAbs (NR-48597); anti-H7 HA pAbs (NR-48597). M2e peptide and stalk peptide aa74-98 were generated by GenScript. In brief, *E. coli* codon-optimized consensus full-length stalk protein (without M2e) was expressed in pGE-RID4 vector and BL21 star (DE3) pLysS cells, and the recombinant stalk protein was purified and used as a control stalk protein^42^.

### Ethics statement

The study in BALB/c mice was carried out in accordance with the recommendations in the Guide for the Care and Use of Laboratory Animals of the National Institutes of Health. Mice were maintained in an animal facility at Georgia State University, USA. All the mouse experiments were approved by Georgia State University Institutional Animal Care and Use Committee (IACUC A21004)

### Immunization and virus challenge studies

BALB/c mice (Female, 6 to 8 weeks old) were intramuscularly (i.m.) immunized in a prime-boost schedule (3-week interval) in the hind legs. M2e-H3 stalk proteins (5–20 μg) were used for prime with adjuvants [10 μg QS-21 (cat no: 34-6-07-2, Desert King International) plus 1 μg monophosphoryl lipid A (MPL, cat no: L6895, Sigma Aldrich)] and boost with half dose adjuvant (5 μg QS-21 + 0.5 μg MPL). For aged mice, 16-month-old BALB/c mice were i.m. immunized. Blood samples were collected after 2 weeks of prime and boost immunization. Four to eight weeks after boost, mice were challenged intranasally with a lethal dose of influenza A viruses. Weight loss of >20% was considered as the IACUC endpoint. Group 1 influenza A viruses were as follows: A/Puerto Rico/8/1934 H1N1 (A/PR8/H1N1), A/California/04/2009 H1N1 (A/Cal/H1N1), A/WSN/1933 H1N1 (A/WSN/H1N1), mouse-adapted A/Fort Monmouth/1/1947 H1N1 (A/FM/H1N1), and reverse genetic (rg) reassortant A/Vietnam/1203/2004 H5N1 with A/PR8 backbone (A/Viet/H5N1), and A/Hong Kong/1073/1999 H9N2 (A/HK/H9N2). Group 2 viruses used include A/Philippine/2/1982 H3N2 (A/Phil/H3N2), A/Hong Kong/1/1968 H3N2 (A/HK/H3N2), reassortants A/Shanghai/11/2013 H7N9 with A/PR8 backbone (A/Sha/H7N9), and A/Nanchang/933/1995 H3N2 with A/PR8 backbone (A/Nanchang/H3N2).

### Enzyme linked immunosorbent assay (ELISA)

M2e-H3 stalk protein vaccine antigenicity using antibodies (1:2000x) specific for known epitopes, IgG antibody responses in sera and in vitro cultures were determined by standard ELISA using M2e peptide (100 ng) M2e-H3 stalk or stalk protein (50 ng), H3 stalk peptides (100 ng), or inactivated viruses (200 ng) as coating antigens. To evaluate antigenicity, the coated antigen was incubated with gene specific primary antibody (1:2000x) and horseradish peroxidase (HRP) conjugated goat anti-mouse IgG (1:2000x) as a secondary antibody followed by tetramethylbenzidine (TMB) substrate (Cat no: 00-4201, Invitrogen) and 1M H_3_PO_4_ to stop the color reaction. The IgG antibody responses or IgG subtypes in sera were determined by using 10-fold serially diluted mice sera and HRP-conjugated goat anti-mouse IgG (Cat no:1030-05, Southern Biotech) IgG1(Cat no:1070-05, Southern Biotech), or IgG2a (Cat no:1080-05, Southern Biotech) as secondary antibodies to determine total IgG or IgG isotype antibodies. The optical density at 450 nm was read using an ELISA reader^14^.

### Determination of lung viral titers

The lung tissues collected day 5 or 6 post infection were homogenized, serially diluted (10-fold) lung extracts (200 µl) injected into 10-day-old embryonated chicken eggs, and then incubated for 3 days at 37 °C. The viral titers were presented in median embryo infectious dose (EID_50_) by hemagglutination assay in allantois fluids. The median embryo infectious dose (EID_50_) was calculated based on the Reed and Muench method^14^.

### In vitro detection of IgG antibody

Mediastinal lymph node (MLN) and spleen cells were isolated at 5 days post infection from young adult (6-week-old) BALB/c mice, immunized and challenged with A/Nanchang/H3N2. The cells were cultured on the plates pre-coated with antigens (M2e peptide, M2e-H3 stalk, or stalk protein for 24 h or 5 days at 37 °C). ELISA was used to determine the IgG antibody titers; the total amount of IgG antibody was quantified based on the standard curve using different concentrations of purified standard IgG (Cat no 1010-01, Southern Biotech)^43,44^.

### Interferon-gamma (IFN-γ) ELISpot

The IFN-γ^+^ secreting cells were evaluated using an ELISpot assay. Briefly, splenocytes and lung cells were isolated 4 weeks after boost immunization and challenged with A/Nanchang/H3N2 virus. IFN-γ^+^ secreting cells were evaluated on the 96-well ELISpot plates pre-coated with 150 ng of IFN-γ-capture antibody (Cat. no. 551216, BD Pharmingen). ELISpot plates were seeded with splenocytes (5 × 10^5^ cells) or lung cells (2 × 10^5^ cells) and incubated with the stimulators: M2e peptide (400 ng), stalk protein (200 ng). After 72 h, the plates were incubated with a biotinylated mouse anti-IFN-γ antibody (1:1000x, cat. no. 554410, BD Pharmingen) followed by alkaline phosphatase-labeled streptavidin antibody (1:1000x, cat. no. 554066, BD Pharmingen), and the IFN-γ-secreting cell spots were visualized with color-developing 3,3'-diaminobenzidine substrates and counted by an ELISPOT reader^14^.

### Flow cytometry

The lung and BALF cell samples were harvested at 6 days post infection from the mice immunized and challenged with A/Nanchang/H3N2 virus. The isolated lung and BALF cells were in vitro culture-stimulated with 5 µg/ml of M2e peptide and/or stalk protein with Golgi stopper (2 µg/mL). After 5 h culture, the cells were stained with mouse anti-CD3-PacificBlue (300x dilution, cat no: 100214, clone 17A2, BioLegend), anti-CD4-biotin (300x dilution, cat no: 13-0042-85, clone RM4-5, eBioscience), streptavidin-PE/Cy5 (300x dilution, cat no: 15-4317-82, eBioscience), and anti-CD8-FITC (300x dilution, cat no: MA5-16759, clone 53–6.7, eBiosciences), followed by fixation and permeabilization using a Cytofix/Cytoperm kit (10x dilution, cat no: 554715, BD Biosciences) and then, staining of intracellular cytokine using IFN-γ mAb (anti-mouse IFN-γ-PE, 200x dilution, cat no: 505808, clone XMG1.2, BioLegend). The IFN-γ^+^ T-cells were acquired by Becton-Dickinson LSR-II/Fortessa flow cytometer (BD) and analyzed by a sequential lymphocyte gating strategy (Supplementary Fig. S10) using FlowJo software (FlowJo V10, Tree Star, Inc.)^45^.

### In vivo protection of immune sera and in vivo T-cell depletion

Mock sera or antisera of M2e-H3 stalk vaccination were heat-inactivated, diluted (4-fold), and mixed with a lethal dose of A/Sha/H7N9 or A/WSN/H1N1 virus. Body weight and survival rates were monitored after intranasal inoculation of naive mice with the mixture of virus and antisera. T-cell depletion in mice was performed with anti-CD4 (GK1.5)cat no: BE0003-1 and anti-CD8 (53.6.7) mAbs (Cat no BE0061 BioXCell)^46^, a day before challenge, 200 µg anti-CD4 mAb and 150 µg anti-CD8 mAb intraperitoneally; a day after challenge, 15 µg anti-CD4 and 10 µg anti-CD8 intranasally. The levels of CD4 and CD8 T-cells were determined by flow cytometry.

### Cell surface ELISA and antibody-dependent cell-mediated cytotoxicity (ADCC) assay

The MDCK cells were seeded on the 96-well cell culture plates (3x10^4^ cells) and then infected with group 1 and 2 influenza A viruses. After 24 h of infection, the virus-infected MDCK cells were fixed with 4% paraformaldehyde and incubated with diluted sera, followed by ELISA to determine IgG antibodies bound to the viral antigens on the MDCK cell surfaces. M2e-specific antibodies in antisera of adjuvanted M2e-H3 stalk vaccination were depleted by serially repeat incubation on the plate coated with M2e peptide antigens to prepare M2e-antibody deficient M2e-H3 stalk antisera for a control ADCC assay. The ADCC Reporter Kit (cat no:M1215, Promega Life Sciences, USA) was used to measure the activation of Jurkat cells mimicking natural killer cells^47^ as a surrogate indicator for ADCC by serum antibodies bound to the virus-infected MDCK cells (3 × 10^4^ cells). After incubation with Jurkat (7 × 10^4^) effector cells for 6 h, luminescence was read to calculate fold increases using a Cytation five imaging reader (BioTek).

### Statistical analyses

Data analyses were performed using Prism software (GraphPad Software Inc). The statistical significance was determined by either one- or two-way ANOVA followed by Tukey’s multiple comparison or Bonferroni post-test. All the data were represented as the mean ± the standard errors of the mean (SEM). *P*-values < 0.05 (*p* < 0.05) was considered statistically significant.

### Reporting summary

Further information on research design is available in the Nature Research Reporting Summary linked to this article.

## Supplementary information

Final revision Supplemental npjvaccines-01942R2

REPORTING SUMMARY

## Data Availability

Data supporting the findings are available from the corresponding authors upon reasonable request.

## Materials & correspondence

Data supporting the findings are unconditionally available from the corresponding author upon request.
