# Supplementary material for: A chimeric thermostable M2e and H3 stalk-based universal influenza A virus vaccine
Source: NPJ Vaccines. 2022 Jun 29;7:68. doi: 10.1038/s41541-022-00498-6 (PMC9243060; doi:10.1038/s41541-022-00498-6)
Supplement: Supplementary file 1 — Final revision Supplemental npjvaccines-01942R2 [file 41541_2022_498_MOESM1_ESM.pdf]

A chimeric thermostable M2e and H3 stalk-based universal influenza A virus vaccine

Jeeva Subbiah[1], Judy Oh[1], Ki-Hye Kim[1], Chong Hyun Shin[1], Bo Ryoung Park[1], Noopur Bhatnagar[1], Baik Lin Seong[2, 3], Bao-Zhong Wang[1], Sang-Moo Kang[1*]

Supplementary information: Supplementary Figures: S1-S11.

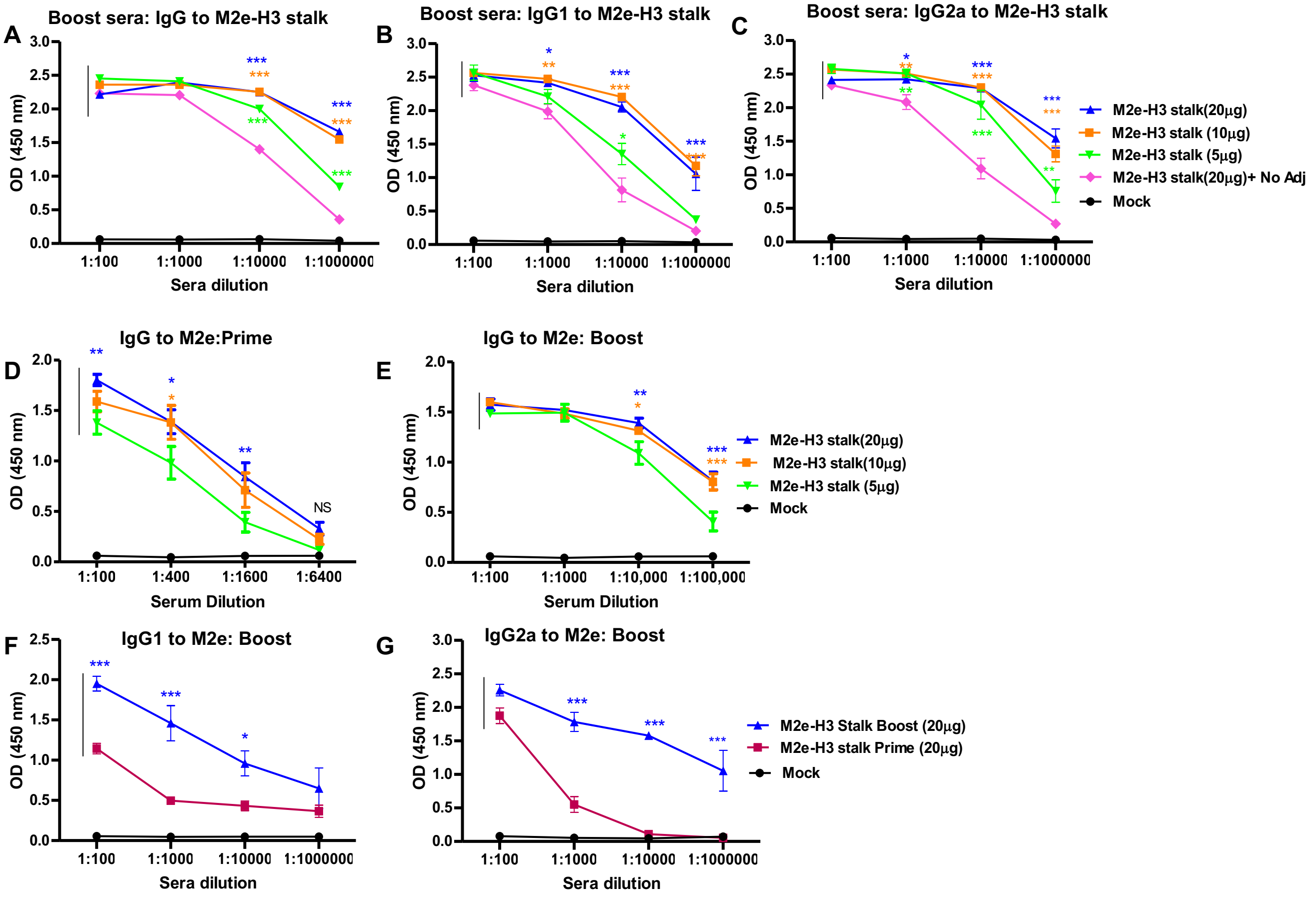

Supplementary Fig S1. Adjuvant and M2e-H3 stalk vaccine dosage effects on inducing IgG antibodies specific for vaccine antigen and M2e.

The levels of IgG, IgG1 and IgG2a antibodies specific for M2e-H3 stalk vaccine antigen (A-C) or M2e (D-G) were measured from prime or boost immune sera collected at two weeks after prime and boost vaccination of young adult mice (n=10) with M2e-H3 stalk protein (5, 10, 20 µg) with adjuvant (QS-21+MPL) or M2e-H3 stalk protein (20 µg) without adjuvant. Mock: adjuvant only naïve sera, No Adj: M2e-H3 stalk vaccinated group without adjuvants. Statistical significance was determined using the two-way ANOVA followed by Bonferroni post-test. Error bars indicate means ± SEM, *, $P < 0.05$; **, $P < 0.01$; ***, $P < 0.001$.

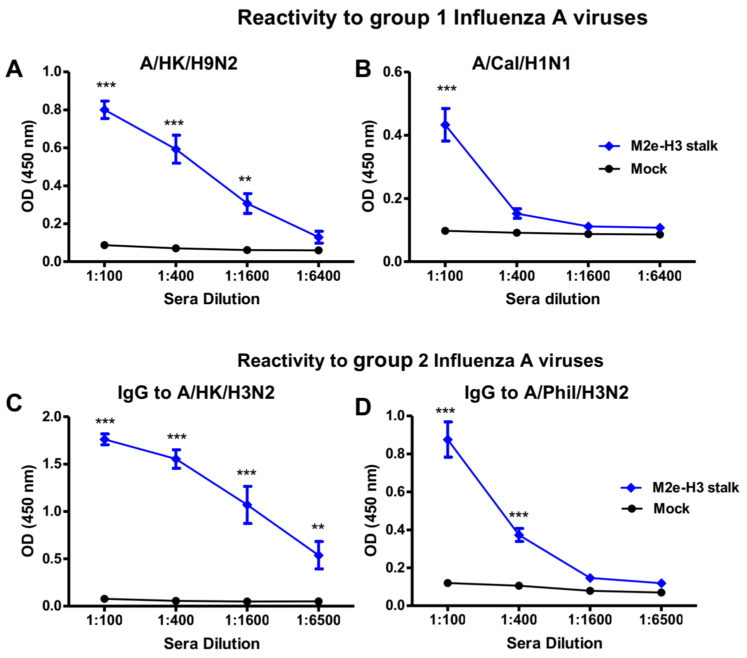

Supplementary Fig S2. Adjuvanted M2e-H3 stalk vaccination induces IgG antibodies recognizing group 1 and group 2 viruses.

Boost sera of M2e-H3 stalk vaccination were used to determine IgG antibodies specific for group 1 viruses, A/HK/H9N2 (A), A/Cal/H1N1 (B) and group 2 viruses, A/Hong Kong (HK)/H3N2 (C), A/Phil/H3N2 (D). Statistical significance was determined using the two-way ANOVA followed by Bonferroni post-test. Error bars indicate means ± SEM, *, $P < 0.05$; **, $P < 0.01$; ***, $P < 0.001$.

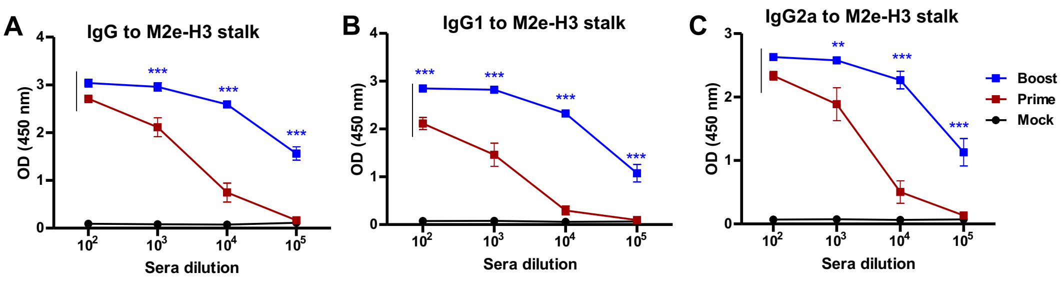

Supplementary Fig S3. Aged mice with adjuvanted M2e-H3 stalk vaccination induce high levels of IgG antibodies specific for vaccine antigen.

Aged BALB/c mice (16 months old) were i.m. prime-boost vaccinated with adjuvanted M2e-H3 stalk and vaccine antigen specific IgG and isotype antibody levels were analyzed in prime and boost sera. IgG (A), IgG1(B), and IgG 2a (C) antibody responses to M2e-H3 stalk protein. Statistical significance was determined between prime and boost groups using the two-way ANOVA. Error bars indicate means ± SEM, *, $P < 0.05$; **, $P < 0.01$; ***, $P < 0.001$.

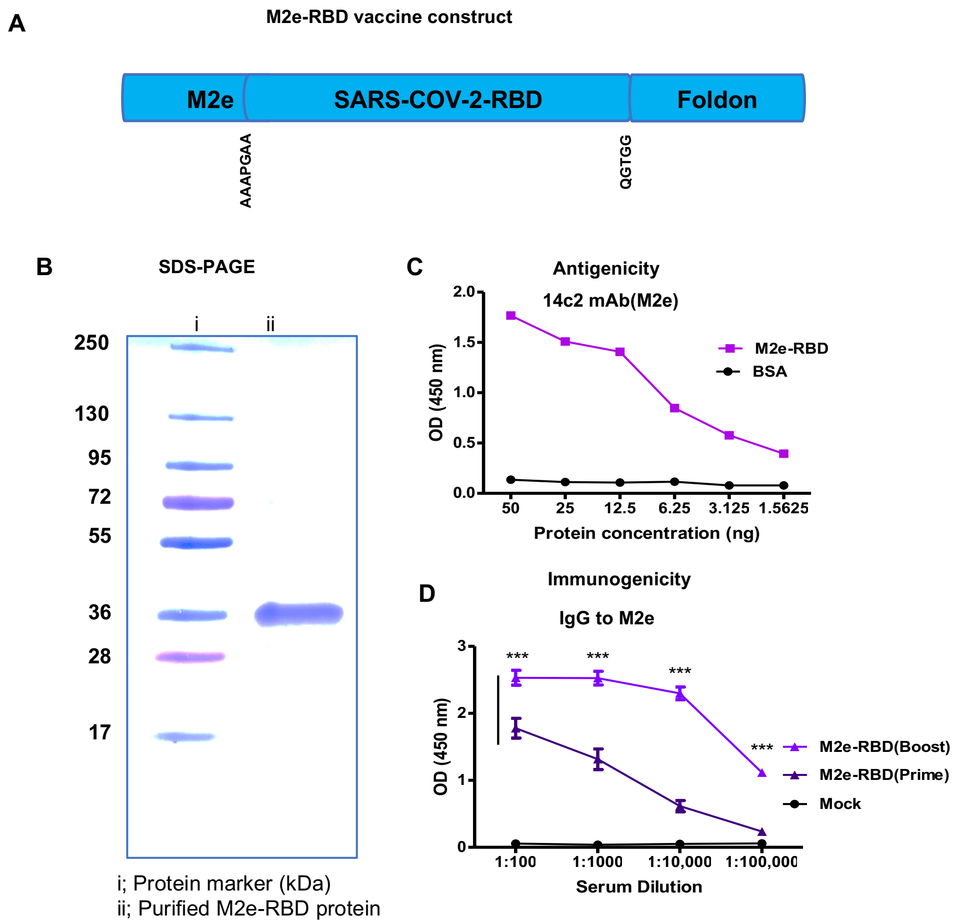

Supplementary Fig S4. Characterization of M2e-RBD (M2e only) vaccine.

(A) Schematic diagram of M2e-RBD (receptor binding domain of SARS-CoV-2 spike protein) vaccine construct. (B) SDS-PAGE gel analysis of the purified M2e-RBD protein. (C) M2e-RBD antigenic reactivity to M2e specific mAb (14C2). (D) IgG antibody of M2e-RBD reactivity to M2e epitope. Statistical significance was determined between prime and boost groups using the two-way ANOVA followed by Bonferroni post-test. Error bars indicate means ± SEM, *, $P < 0.05$; **, $P < 0.01$; ***, $P < 0.001$.

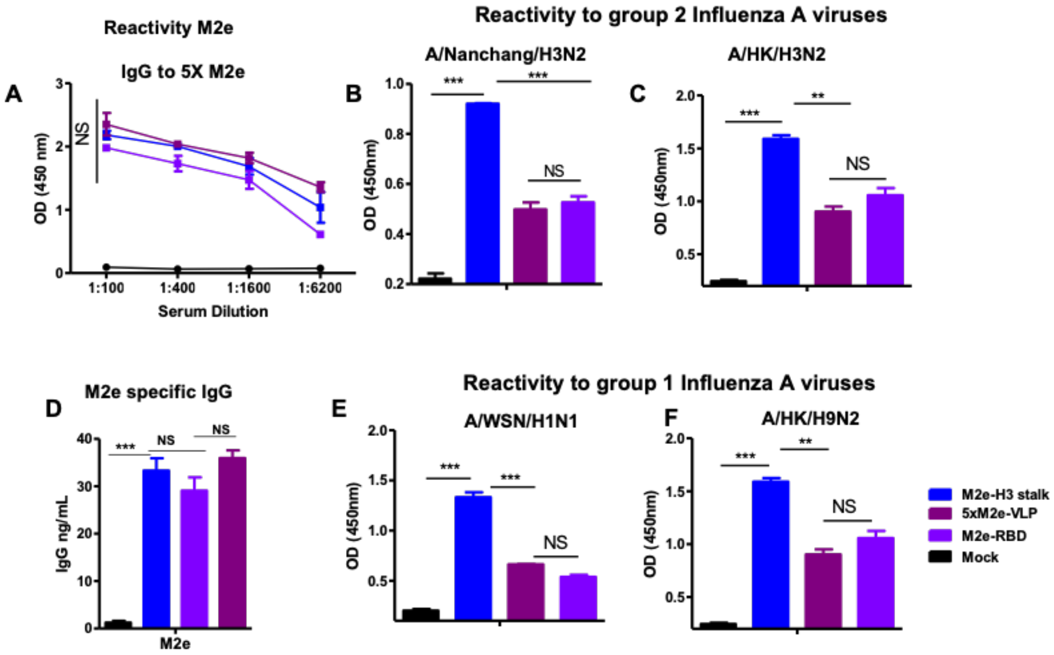

Supplementary Fig S5. Adjuvanted M2e-H3 stalk vaccination induced higher levels of IgG antibodies binding to viral antigens on the influenza A virus infected MDCK cell surface than M2e only vaccines.

Boost antisera of M2e-H3 stalk and M2e only vaccination was compared in the levels of IgG antibodies specific for 5xM2e repeat protein and virus infected cell surface viral antigens. (A) IgG antibodies for 5xM2e. (B) A/Nanchang H3N2 virus infected MDCK cell surface ELISA. (C) A/Hong Kong H3N2 virus infected MDCK cell surface ELISA. (D) M2e specific IgG quantification between the groups from panel A. (E) A/WSN H1N1 virus infected MDCK cell surface ELISA. (F) A/HK H9N2 virus infected MDCK cell surface ELISA. Statistical significance was determined using the one or two-way ANOVA. Error bars indicate means ± SEM, *, $P < 0.05$; **, $P < 0.01$; ***, $P < 0.001$.

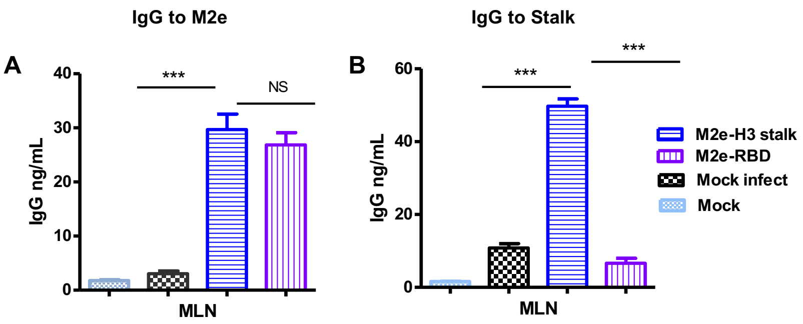

Supplementary Fig S6. Adjuvanted M2e-H3 stalk vaccination induces the generation of stalk-specific IgG antibody-secreting plasma cells but not M2e-RBD.

Mediastinal lymph nodes (MLN) were collected at 6 days-post-infection with A/Nanchang H3N2 virus from adjuvanted M2e-RBD (20µg) or M2e-H3 stalk (20µg) vaccinated young adult mice. *In vitro* production of IgG antibodies specific for M2e (A) and stalk (B) was assessed in MLN cultures. Mock: adjuvant only naïve sera; mock inf: mock group with virus infection. Statistical significance was determined using the one-way ANOVA. Error bars indicate means ± SEM, *, $P < 0.05$; **, $P < 0.01$; ***, $P < 0.001$.

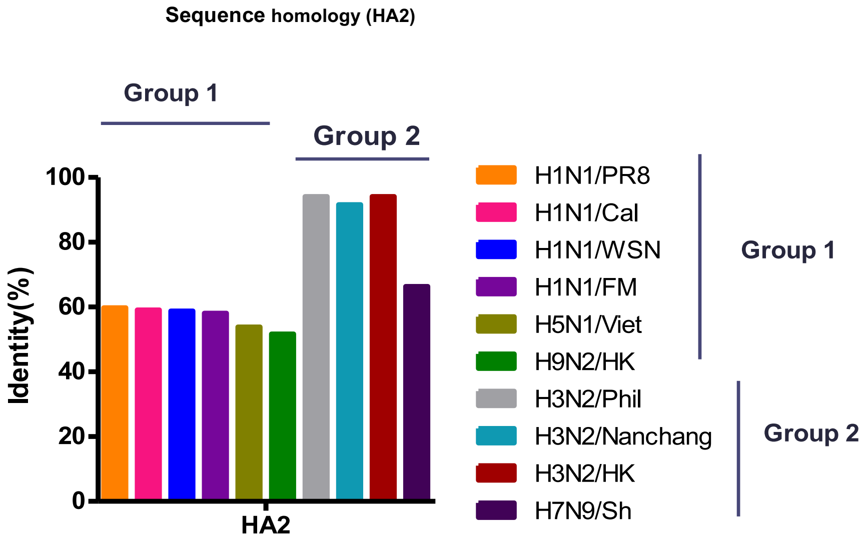

Supplementary Fig. S7. Analysis results of amino acid sequence homology among M2e-H3 stalk and its corresponding domain of group 1 and 2 challenge viruses.

The HA2 domain sequences of the challenging viruses used in this study were obtained from GenBank to analyze the identity of amino acid (aa) sequences: GenBank accession No: NC_002017 H1 HA (YP_163736) for A/Puerto Rico/8/1934 (H1N1/PR8); CY010788 H1 HA (ABF47955) for A/WSN/1933 (H1N1/WSN); IVU02464 H1 HA (AAC53844) for A/Fort Monmouth/1/1947 (H1N1/FM); NC_026433A H1 HA (YP_009118626) for A/California/07/2009 (H1N1/Cal); EU122404 H5 HA (ABW90135) for A/Vietnam/1203/2004 (H5N1/Vet); KF188366 H9 HA (AGO17823) for A/chicken/Hong Kong/G9/1997 (H9N2/HK); IVU08858 H3 HA (AAA18781) for A/Philippines/2/82 (H3N2/Phil); KC853228 H7 HA (AGI60292) for A/Shanghai/2013 (H7N9/Sh); CY080523 H3 HA (ADV76673) for A/Hong Kong/1-10-MA21-1/1968 (H3N2/HK); KM821307 H3 HA (AIU46048) for A/Nanchang/933/1995 (H3N2/Nanchang). The percentages of the aa homology of the vaccine construct M2e-H3 stalk HA1 and HA2 domains with HA sequences of challenge viruses were calculated using Needle program (EMBOSS; EMBL-EBI).

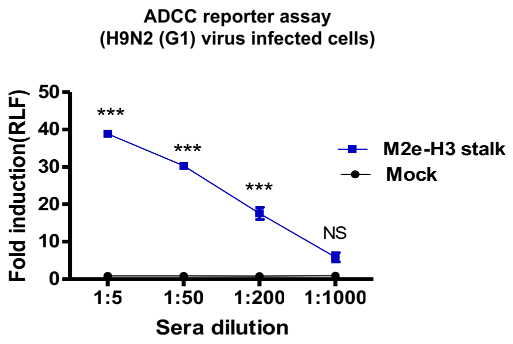

Supplementary Fig. S8. ADCC reporter assay activity on H9N2 virus infected MDCK cells. The boost sera of adjuvanted M2e-H3 stalk were used to determine the ADCC reporter assay activity using A/HK/H9N2 infected MDCK cells. Statistical significance was determined using the two-way ANOVA followed by Bonferroni post-test. Error bars indicate means ± SEM, *, $P < 0.05$; **, $P < 0.01$; ***, $P < 0.001$.

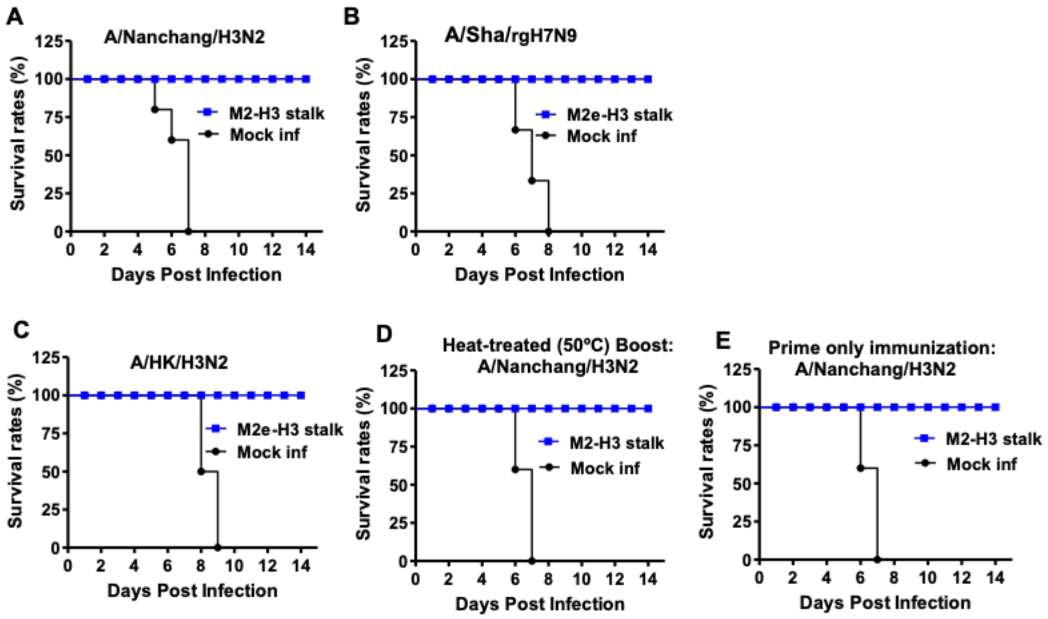

Supplementary Fig. S9 Adjuvanted M2e-H3 stalk vaccinated mice survived 100% against group 2 influenza A virus infection.

The survival rates of adjuvanted M2e-H3 stalk groups were determined against group 2 influenza A virus infection from Figs 4C-H.

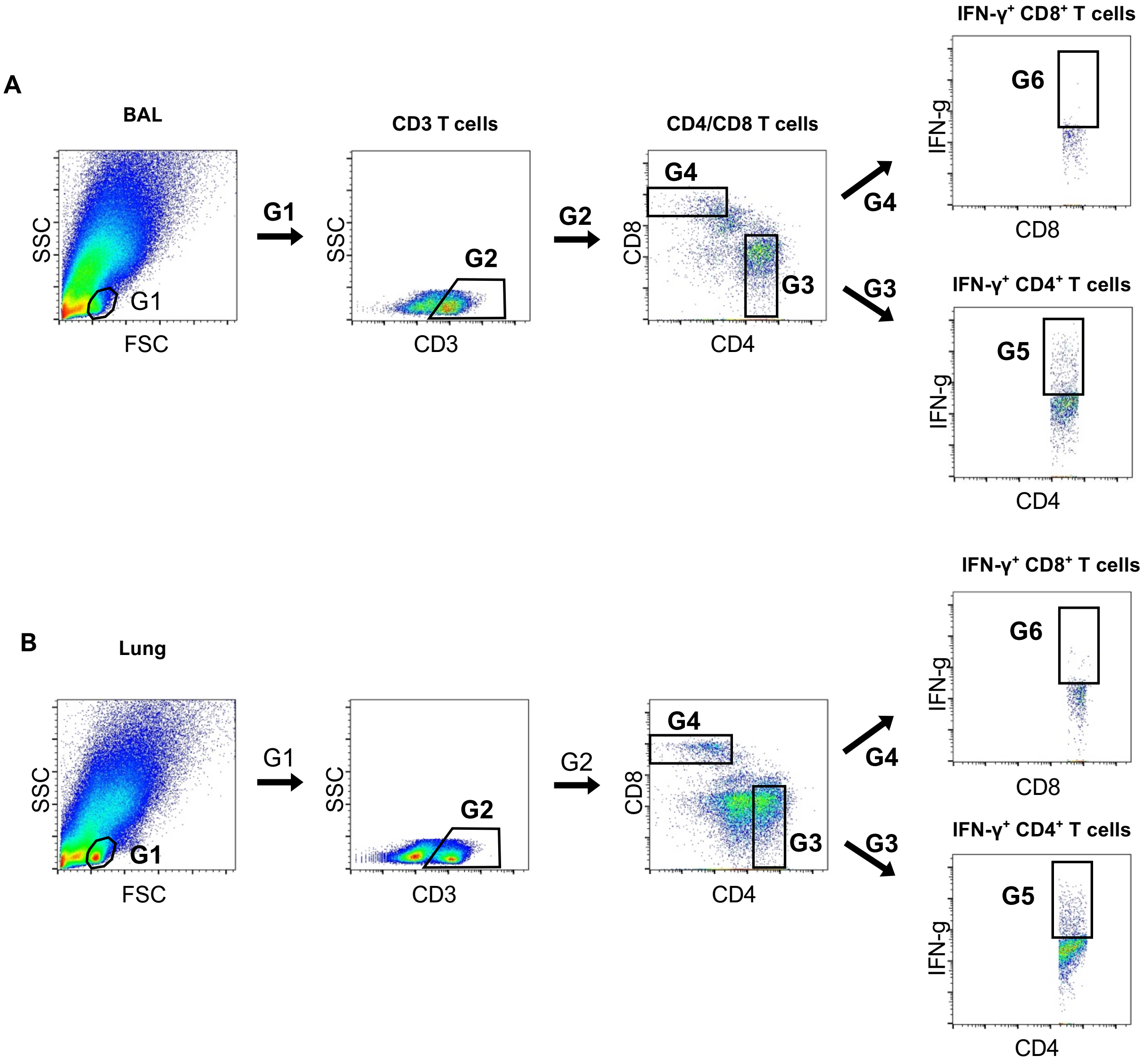

Supplementary Fig. S10. Flow cytometry analysis to identify CD4 and CD8 T cells secreting IFN-g after influenza virus infection.

The effector T cells were analyzed in BAL (A) and lung cells (B) obtained from mice with immunization and challenge by flow cytometry. G1: lymphocytes gated from total cells, G2: $CD3^+$ T cells gated from lymphocytes (G1), G3 and G4: $CD4^+CD3^+$ T cells or $CD8^+CD3^+$ T cells gated from $CD3^+$ T cells (G2) respectively, G5: IFN-g^+CD4^+ T cells gated from $CD4^+CD3^+$ T cells (G3), G6: IFN-g^+CD8^+ T cells gated from $CD8^+CD3^+$ T cells (G4).

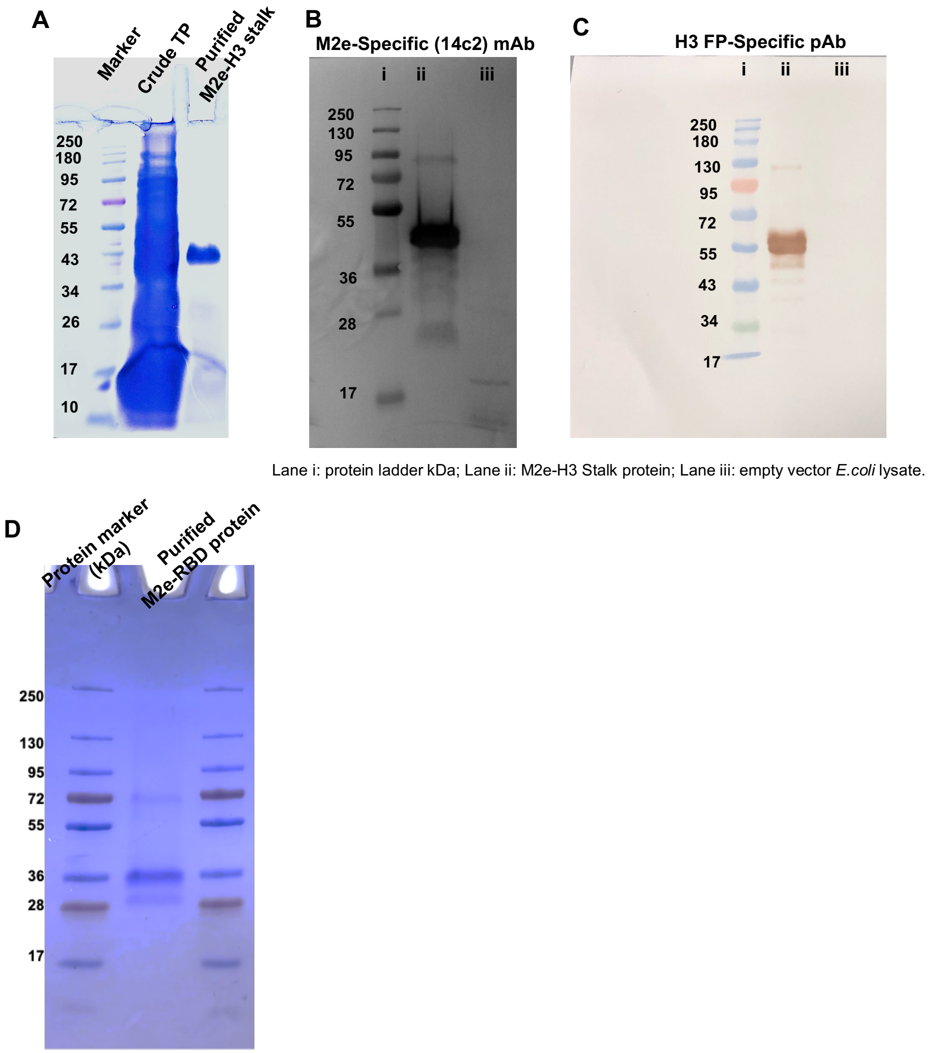

Supplementary Fig. S11. SDS PAGE and Western blot analysis.

All the purified proteins and western blots were derived from the same experiment and processed in parallel. (A) Purified M2e-H3 stalk protein. (B) M2e specific western blot against M2e-H3 stalk. (C) H3 FP-specific western blot against M2e-H3 stalk protein. (D) Purified M2e-RBD protein.
